# Integration of Green Energy and Advanced Energy-Efficient Technologies for Municipal Wastewater Treatment Plants

**DOI:** 10.3390/ijerph16071282

**Published:** 2019-04-10

**Authors:** Ziyang Guo, Yongjun Sun, Shu-Yuan Pan, Pen-Chi Chiang

**Affiliations:** 1 Graduate Institute of Environmental Engineering, National Taiwan University, Taipei City 10673, Taiwan; zyguo93@hotmail.com; 2 Carbon Cycle Research Center, National Taiwan University, Taipei City 10672, Taiwan; 3 College of Urban Construction, Nanjing Tech University, Nanjing 211800, China; 4 Department of Bioenvironmental System Engineering, National Taiwan University, Taipei City 10617, Taiwan; 5 Energy Technologies Area, Lawrence Berkeley National Laboratory, Berkeley, CA 94720, USA

**Keywords:** wastewater treatment plant, green energy, advanced energy-efficient technologies, key performance indicators

## Abstract

Wastewater treatment can consume a large amount of energy to meet discharge standards. However, wastewater also contains resources which could be recovered for secondary uses under proper treatment. Hence, the goal of this paper is to review the available green energy and biomass energy that can be utilized in wastewater treatment plants. Comprehensive elucidation of energy-efficient technologies for wastewater treatment plants are revealed. For these energy-efficient technologies, this review provides an introduction and current application status of these technologies as well as key performance indicators for the integration of green energy and energy-efficient technologies. There are several assessment perspectives summarized in the evaluation of the integration of green energy and energy-efficient technologies in wastewater treatment plants. To overcome the challenges in wastewater treatment plants, the Internet of Things (IoT) and green chemistry technologies for the water and energy nexus are proposed. The findings of this review are highly beneficial for the development of green energy and energy-efficient wastewater treatment plants. Future research should investigate the integration of green infrastructure and ecologically advanced treatment technologies to explore the potential benefits and advantages.

## 1. Introduction

### 1.1. Wastewater Treatment

According to the treatment scale, the environmental function of the discharged water body, and the local environmental protection requirements, there are three processes which can be selected for water treatment: the first-level strengthening treatment process, the secondary treatment process, and the secondary strengthening treatment process. First-level strengthening treatments usually utilize materialized strengthening treatment methods [1], such as the pre-stage process of the adsorption biodegradation (AB) method, the pre-stage process of the hydrolysis aerobic process, the high-load activated sludge process, etc. The secondary treatment process can use the activated sludge process, oxidation ditch process, sequencing batch reactors (SBR) process, hydrolysis aerobic method, AB method, and biological filter method [2,3,4]. Secondary strengthening treatment processes can choose the Anoxic Oxic(A/O) method or the Anaerobic-Anoxic-Oxic (A/A/O) method; the goal is to remove carbon source-pollutants while strengthening the function of nitrogen and phosphorus removal [5]. According to present surveys, the most widely used treatment process is the ordinary activated sludge method, including the Anaerobic-Anoxic-Oxic, SBR, and oxidation ditch methods in municipal wastewater treatment plants which are already completed and operating in China [6].

**Figure 1:**
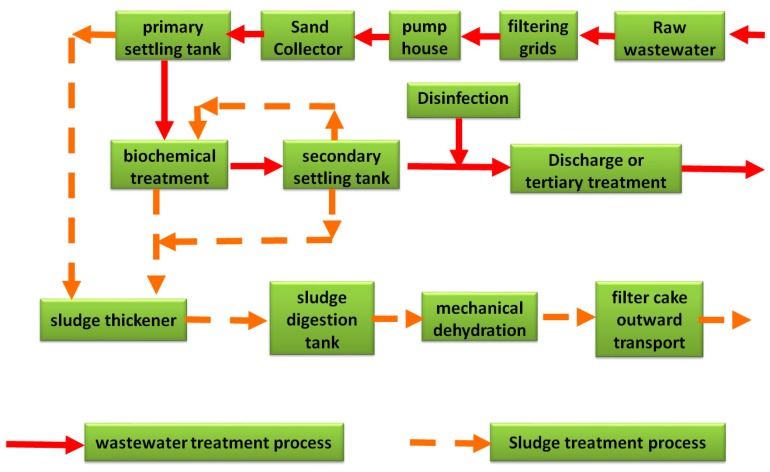
Typical process flow diagram of a wastewater treatment plant (WWTP).

At present, there are dozens of urban sewage treatment plants with a processing scale of more than 200,000 m^3^/day in China, and the most common method applied are the activated sludge method and the improved Anoxic Oxic method and Anaerobic-Anoxic-Oxic method [7]. Large-scale sewage treatment plants in large cities have the advantages of high economic strength, high technical levels, and strong management experience [8]. The larger the scale, the lower the energy consumption and the lower the operating cost. With the development of technology and economic level, the activated sludge process has great potential for developing the collection and utilization of biogas in the anaerobic section [9]. If the biogas generated in the sewage treatment process can be utilized, it would inevitably reduce energy consumption. The process flow for a typical wastewater treatment plant (WWTP) is shown in Figure 1. The solid red arrows indicate the process flow of the wastewater treatment process and the orange dotted arrows indicate the process flow of the sludge treatment process.

Small and medium-sized sewage treatment plants are mainly located in small- and medium-sized cities and small towns. The investment is relatively low, and the number of existing wastewater treatment plants is relatively large [10]. According to current construction examples, the majority of the selected processes are the oxidation ditch method and the SBR method. The oxidation ditch method and the SBR method have the advantages of requiring a primary sedimentation tank and less space, having a relatively strong impact resistance, and a high removal efficiency of organic matter. The processes can meet the nitrogen and phosphorus removal requirements, and the sludge does not need to be digested after aeration treatment. It can be directly used in the fields of fertilization, landfills, and incineration treatment through concentrated dewatering. However, the oxidation system and SBR aeration system lead to higher operating costs, such as electricity consumption. For small- and medium-sized wastewater treatment plants, the oxidation ditch method and the SBR method require 10–15% less investment compared to the activated sludge process [11]. The facilities are simple and easy to manage and operate, which are suitable for economic benefit and local management technology in medium and small cities.

### 1.2. Energy Consumption in WWTPs

**Figure 2:**
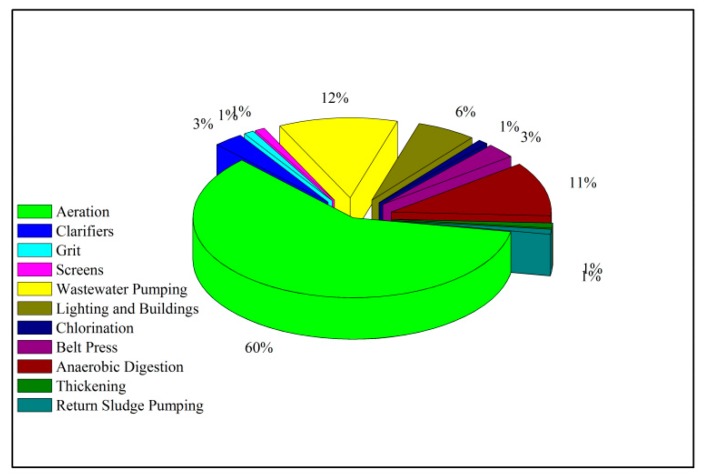
Energy distribution in conventional activated sludge systems [16].

The energy consumption of wastewater treatment plants can be generally classified into direct energy consumption and indirect energy consumption. Direct energy consumption is the electrical energy required for operation of aeration blowers, lift pumps, return pumps, etc. Indirect energy consumption includes chemicals used for chemical phosphorus removal and sludge dewatering [12]. For power consumption of the secondary treatment process, the energy consumption of the sewage lift pump generally accounts for 10–20% of the total energy consumption, biological treatment accounts for 50–70%, sludge treatment and disposal accounts for 10–25%, and the proportion of these three parts take up more than 70% [13]. Figure 2 shows the energy consumption for a conventional activated sludge system, where aeration consumes most of the energy (60%) in the process. Nowadays, with the improvement of emission standards in wastewater treatment, advanced treatment processes have been adopted in many wastewater treatment plants, including denitrification filters, sand filtration, and UV disinfection. Therefore, the lifting pump and blast aeration are the key points for energy savings and consumption reduction in sewage treatment plants. The existing energy savings and consumption reduction methods include improving the existing process or equipment operation and reducing the operating energy consumption [14]. In addition, the energy consumption can also be optimized from the wastewater treatment process, thereby reducing the energy consumption of wastewater treatment plants [15].

### 1.3. Objectives

In this paper, the energy-saving technologies and capacity technologies in wastewater treatment are reviewed and introduced through a literature review, and the green energy sources available in wastewater treatment plants are also introduced. Through the review and summary of this article, it is hoped that the energy savings and technological progress of wastewater treatment plants can be promoted. The main key research contents of this paper are as follows: (1) The implementation of green energy and energy-efficient technologies for wastewater treatment plants are reviewed. Green energy and biomass energy utilized in WWTPs are addressed. (2) The challenges and perspectives of the water and energy nexus for WWTPs are presented. (3) Key performance indicators for the integration of green energy and energy-efficient technologies are developed. Comprehensive elucidation of energy-efficient technologies for WWTPs are revealed. (4) The Internet of Things (IoT) and green chemistry technologies are introduced for system optimization of WWTPs. The water and energy nexus of wastewater treatment plants in China are also revealed and discussed.

## 2. Challenges and Barriers

### 2.1. Confluence System Rainwater and Initial Rainwater

At present, the designed sewage volume of urban sewage treatment plants in China only calculates the amount of sewage and a small amount of groundwater infiltration [17]. The amount of rainwater in the combined system has not been considered, and the runoff rainwater entering the sewage system has also not been considered [18]. However, there are still confluence areas in some parts of China, even if the area was built according to the diversion system. Some sewage treatment plants in the rainy season exceed the treatment capacity, which will cause the deterioration of environmental quality when excess sewage and rainwater overflows into the water environment.

### 2.2. Low Influent Concentrations

According to the chemical oxygen demand (COD) analysis results of sewage treatment plant influents, the influent COD of urban sewage treatment plants in China is low. The main reasons are as follows: (1) Part of the sewage treatment plant is not complete. Sewage pipe networks are affected by various factors during operation, such as ruptures, leakages, and staggering. (2) In the areas with high groundwater levels, the above factors would cause infiltration of groundwater and dilution of sewage [19].

### 2.3. Low Sewage Recycling Ratio

Many cities are lacking in water safety to drink and the ecological environmental water cannot be guaranteed. In addition, the tail water of sewage treatment plants that have been treated with high standards cannot be effectively utilized. In this case, the reclaimed water has become a veritable second water source. In Beijing, there is more than 1 billion m^3^ of reclaimed water which is being used every year [20]. At present, the reclaimed water should be investigated especially in water-deficient areas, then, practical and feasible countermeasures to ensure reclaimed water can be used as a second water source should be proposed [21].

### 2.4. Unsafe Sludge Disposal

Large amounts of sludge are not being stabilized in sewage treatment plants, and they are potentially dangerous secondary sources of pollution in the transportation and disposal processes. Unsafe sludge disposal would largely negatively affect the environmental benefits from sewage treatment facilities [22]. Sludge treatment is as important as water treatment. The proper evaluation of investment and disposal costs is necessary to strengthen coordination among various departments and industries [23].

### 2.5. Low Energy Efficient

It has been proposed to construct an energy self-sufficient sewage treatment plant that is carbon neutral [24]. Administrative departments can achieve energy self-sufficiency by reducing the energy consumption of sewage treatment, utilizing exogenous organic matter and sludge, improving the synergism of high-efficiency anaerobic digestion and cogeneration. At present, however, most construction units in urban sewage treatment plants eliminate the anaerobic digestion of sludge during the feasibility stage due to the complexity of anaerobic management and low organic matter content of sludge [25]. In recent years, the construction and operation of urban sewage treatment plants should improve the energy self-sufficiency rate of sewage treatment plants through high-efficiency anaerobic digestion and synergistic anaerobic digestion technology on the basis of reducing the electricity consumption per ton of sewage treatment [26].

## 3. Green Energy for Wastewater Treatment Plants

### 3.1. Solar Energy

**Figure 3:**
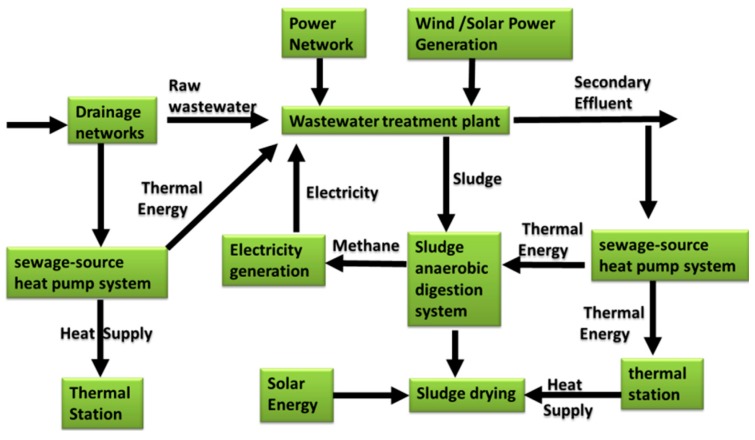
Energy utilization diagram of a wastewater treatment plant.

With the operation of a large number of sewage treatment plants, the problem of sludge disposal has become an imminent and difficult problem in the water treatment industry and the social environment [27]. In order to reduce the cost of sludge disposal, the sludge must first be dewatered to reduce the volume. Thermal drying technology with a combustion process can be used to further reduce the sludge volume after reaching the limit of mechanical dewatering (50–60% dry matter) [28]. Solar energy, which is considered the most abundant renewable energy source, can be introduced into WWTPs. Figure 3 shows the utilization of solar energy in the wastewater treatment process. The application of solar thermal energy in wastewater treatment mainly includes three aspects: (a) the solar heat is collected through a heat collector to increase the reaction temperature and improve the treatment efficiency [29]; (b) the solar thermal is used to dewater the sludge or reduce the water content of some special wastewater in industrial wastewater treatment [30]; and (c) the solar heat can be used for evaporation and desalination of special wastewater in industrial wastewater treatment [31]. The application of solar photovoltaic in wastewater treatment mainly includes two aspects: (a) the pollutant can be removed and recovered through photovoltaic power generation electrolysis; and (b) the solar photovoltaic can provide electricity for sewage biological treatment through photovoltaic power generation [32].

#### 3.1.1. Photothermal Utilization

(a) Wastewater and Anaerobic Treatment System Heated by Solar Energy

There are enough solar energy resources in Northern China to use solar energy instead of traditional energy. Solar energy is able to increase the anaerobic treatment temperature of sewage to achieve higher sewage treatment efficiency. Based on the design of solar energy anaerobic wastewater treatment systems, it can solve the energy problem in sewage anaerobic treatment heating systems for the solar energy-rich areas. The use of solar pre-heated sewage in anaerobic pools can solve the low temperature and freezing problems of wastewater, which are caused by the large temperature differences in plateau areas. Yiannopoulos [33] used Anaerobic Biofilter reactors to increase the sewage treatment temperature by solar heating. The results show that solar heating can make the temperature meet the intermediate temperature (35 °C) of anaerobic reactions. This indicates solar heating anaerobic biological treatment has good application prospects. Ren [34] summarizes the technical methods of solar wastewater treatment systems, and proposes that the improvement of anaerobic biological treatment should be carried out from the development and utilization of solar heating systems.

(b) Sludge Drying Technology by Greenhouse-Type Solar Energy

A greenhouse-type solar drying device has the advantages of a simple structure and easy operation. In order to obtain more solar radiation, materials used on the drying device usually have high transmittance. However, a large enough space is needed for the construction of the drying chamber to make sure sludge can receive enough sun radiation to reach low water content. Luboschik [35] already began research on sludge solar drying in 1994. A drying test done in Southern Germany showed that the annual evaporation of water per unit area can reach 700–800 kg, and recycling waste heat can increase the coefficient of performance by 3–4 times.

(c) Heat Collector and Storage Solar Sludge Drying Technology

In order to improve the stability of the drying system, a collector solar drying system can be used. The solar collector is used to convert solar energy into electricity which is able to heat water with a constant water temperature, so that the sludge can be thermally dried by hot water through floor radiation [36]. Mathioudakis et al. [37] studied the drying of sludge in Greece using a heat collecting and storage solar drying device. The results show that the sludge moisture content needed to be reduced from 85–6% for 7–12 days in the summer and reduced to 10% for 9–33 days in the autumn, at the same time, the volume of sludge was reduced to 15–20%. If a solar hot water circulation system was installed at the bottom of the unit for assistance, the sludge drying time could be shortened to 1–9 days even in winter [38].

(d) Heat-Pump Solar Greenhouse Sludge Drying Technology

Sludge drying technology combining solar energy and a heat pump fully utilizes the heat-pump system to reach high evaporation temperatures with high efficiency. A solar collector has the advantages of good heat collecting performance at low temperatures [39]. Through this new combination system, it effectively overcomes the instability problem of solar energy. At same time, it achieves the goal of energy saving, emissions reduction, and pollution prevention [40]. Slim et al. [41] carried out a drying study using a heat-pump solar greenhouse sludge drying system, and the results show that the increasing temperature of heated air can accelerate the evaporation of water under the fixed heating temperature conditions.

#### 3.1.2. Photoelectric Utilization

Some researchers have suggested that photovoltaic power generation that provides electricity for sewage biological treatment can reduce the energy consumption of sewage treatment plants [42]. Sewage treatment plants have a high electric load with large energy consumption, and they are operated continuously for 24 h with a stable load [43]. The power generated from a photovoltaic power station can satisfy the needs of a water treatment plant, which is in line with the “self-sufficiency” mode [44]. The electricity consumption takes up more than 30% of production costs, so cost reduction and efficient treatment is required intensively [45]. The combination of photovoltaic power and wastewater treatment with the implementation of contract energy management can further reduce the cost of wastewater treatment. At present, it also has some application cases for treating sewage using solar power as a power source. Hudnell et al. [46] adopted solar-powered circulating agitation technology to replace conventional aeration technology of oxidation ponds, which can effectively reduce the electricity consumption and operating costs of the oxidation pond, while also deodorizing and reducing sludge production and greenhouse gas emissions. Han et al. applied solar power to drive the oxidation ditch without the battery, and the solar power system automatically starts and stops depending on the change in light intensity [47].

#### 3.1.3. Photocatalysis

The basic principle of solar photocatalysis is that by exciting the electrons in the semiconductor, the electrons are excited from the valence band to the conduction band to generate photogenerated electrons, thereby generating photogenerated holes in the valence band, and the electrons and holes are respectively diffused to the semiconductor surface [48]. The surface reacts with different organic matters to degrade the contaminates. Some organic or inorganic pollutants in the sewage are detoxified by an oxidation-reduction reaction. Photocatalysis has its unique advantages of rapid reaction, complete oxidation, no secondary pollution, mild reaction conditions, and no need for excessive equipment. It is often used for advanced treatment or wastewater reuse treatment in sewage treatment plants [49].

### 3.2. Wind Energy

Although the distribution of wind energy has certain limitations, it is a widely distributed energy source compared to other renewable energy such as mineral energy, water energy, and geothermal energy. All regions can make rational use of renewable energy sources, like wind and solar conditions. According to local conditions, different wind turbines are used depending on different loads, so that wind energy can be converted into mechanical energy to the maximum extent [50]. The power grid is used as an auxiliary energy source, and it is suitable for the fields of wind energy and sewage treatment plants at various scales. The construction of wind turbines will combine grid power and wind power, which is suitable for areas with a rich wind source [51].

**Figure 4:**
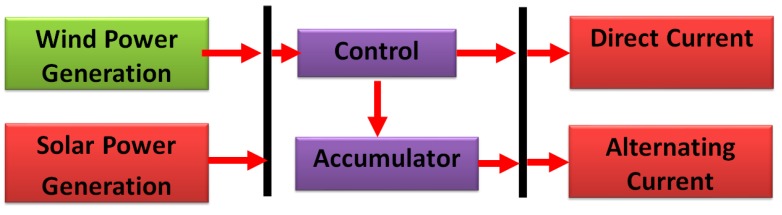
Wind energy and solar energy complementary intelligent system diagram.

Wind energy and solar energy can be applied without emitting pollutants or exhausting gas into the atmosphere [52]. They are environmentally friendly and pollution-free energy sources. Both wind and sunshine intensity constantly vary with weather and climate, which makes wind and solar energy difficult to be used [53]. However, with the development of modern science and technology, the utilization of wind energy and solar energy has made breakthroughs in technology [54]. In particular, the comprehensive utilization of wind energy and solar energy (Figure 4) take full advantage of their complementarity, establishing a more stable, reliable, economical, rational energy system in many aspects [55].

### 3.3. Heat Energy

The sewage source heat pump uses sewage as an energy source to exchange heat between the sewage and the heat pump, and the internal heat pump was derived by electric power to achieve cooling or heating. Urban sewage has the characteristics of large sewage volume, stable water quality, and relatively stable internal temperature [56]. Therefore, the working performance is relatively stable when using the sewage source heat pump system. The energy efficiency ratio of the sewage source heat pump is mainly affected by the water volume and water temperature on the inlet side and the user side. Generally, the heating/cooling coefficient of the sewage source heat pump is 5.0–6.0 [57].

Compared with common air-source heat pumps, sewage-source heat pumps have a higher coefficient of performance [58]. In addition, fossil energy is not required in sewage-source heat pump systems, and does not generate secondary pollution. It is a technically feasible, economically affordable, and environmentally friendly method for comprehensive utilization of urban sewage. Baek et al. [59] found that the sewage source heat pump system can reduce CO_2_ emissions by 68% and SO_2_ emissions by 75% compared with the air-source heat pump system [60]. The secondary effluent of sewage treatment plants used in the sewage-source heat pump conditioning system has many significances [61]. The recovery and reuse of the effluent from a sewage plant can alleviate the current situation of water shortages in China to some extent [62]. Development and utilization of low-level energy sources in sewage through heat-pump technology can replace part of coal-fired and oil-fired boilers, which can properly alleviate environmental problems [63].

## 4. Advanced Energy Efficient Technologies for Wastewater Treatment Plants

### 4.1. Pumps

The influent of the sewage treatment plant is at the end-pipe network system, and it has relatively low elevation. Therefore, it is necessary to use a lifting pump to lift the sewage into the treatment system [64]. This process could be energy intensive, the main reasons for the high energy consumption of the pumps in sewage treatment plants is the incorrect pump design or selection [65]. Energy consumption can be improved by designing proper pumps in a right position. The energy savings of the sewage lifting angle needs to be comprehensively analyzed from the sewage lifting system. First of all, in the design stage of the sewage treatment process, it is necessary to broadly investigate the existing pipe network system and the whole process facilities of sewage treatment to minimize the wastewater elevation that needs to be upgraded with the elevation of the processing facility and consideration of the flooding flow mode [66].

**Figure 5:**
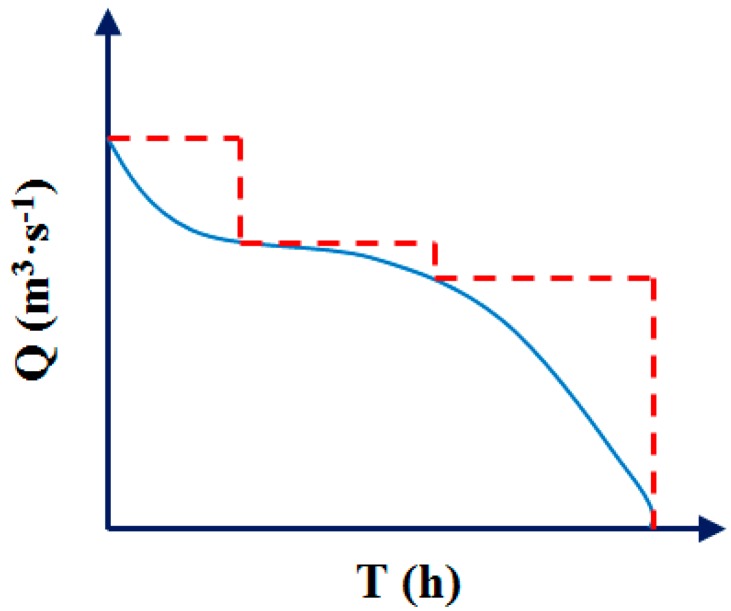
Water pump flux to time according to actual flow rate at different times.

Secondly, it is necessary to select the appropriate pump according to the combination of sewage lifting amount and changing characteristics. According to the variation curve of the pipeline system, especially the sewage flow rate (Figure 5), appropriate pumps are needed to meet the high operating efficiency range and high water level conditions [67]. According to the sewage treatment volume, head, head loss, and pump power, the appropriate and efficient pump combination are selected. This includes setting the ratio and regulation between the variable frequency pump with the frequency converter and the fixed power pump without the frequency converter. The aim of setting is to reduce the pump shaft power and avoid frequent opening of the pump which will reduce the service life of the pumps [68]. Furthermore, they are more focused on the matching performance between the pump and the motor to enhance the efficiency of motor operation. In addition, the pipeline should be well designed to ensure the compact and smooth system for reducing the length of bends and pipelines, and the resistance and energy consumption of pipeline transport systems [69]. Finally, it is necessary to pay attention to process operation management, equipment maintenance, dripping reduction, scaling, and mechanical wear of the operating system. In addition, ensure that equipment and systems operate under high-efficiency conditions [70]. In the design and operation of different wastewater treatment plants, the improvement of the lift pump is achieved mainly by the application of variable frequency control technology.

### 4.2. Aeration

The removal of pollutants in sewage is mainly achieved through microbial biochemical metabolic processes. Wastewater treatment biochemical processes mainly include the Anaerobic-Anoxic-Oxic process, oxidation ditch process, and the SBR process [71]. The process for biochemical metabolism of microorganisms to remove pollutants requires the presence of electron acceptors which is mainly provided by aeration and oxygen supply [72]. Therefore, effective aeration is an important method for pollutant removal and effective sewage treatment. In addition, in the process of pollutant removal, such as denitrification in the A2O process, the mixture reflux is required to provide nitrate nitrogen as an electron acceptor [73]. Chemical agents are required to enhance chemical precipitation in the process of chemical phosphorus removal, which also cause certain energy consumption. Aeration control is the key point for energy savings and consumption reduction in the biological sewage treatment process [74]. The differences in the aeration profiles between traditional control and optimized conditions. Aeration control includes optimization of the aeration device, aeration pipe arrangement, and optimization of aeration supply mode [75].

For aeration methods, the A2O and SBR processes generally use microporous aeration, while oxidation ditch generally uses rotary brush aeration or inverted umbrella aeration [76]. Microporous aeration mainly enhances oxygen transmission efficiency by generating microbubbles with a diameter of 1.5–3.0 mm. However, the microporous aeration process requires high energy due to the large aerodynamic resistance of the air flow through the micropores. Therefore, it is necessary to introduce a stirrer at the bottom of the chamber for microporous aeration; this design has also been applied to many oxidation ditch processes [77]. In the past, it was considered that unilateral aeration could reduce the air volume, but it has been proven that uniform small vortex and local mixing can be formed by comprehensive aeration, which strengthen the transmission of small bubbles and enhances oxygen transmission efficiency.

In addition to aeration devices and aeration methods, the supply of aeration is a key research object for energy conservation and consumption reduction. If the amount of aeration is too small, the quality of the effluent from the sewage treatment would be affected; if the aeration is too large, it will cause waste of energy, change in sludge floc structure, and effect sedimentation of the activated sludge [78]. The core of aeration energy conservation is to provide the required electron acceptor which dissolves oxygen on demand in order to ensure the effective removal of pollutants in the biochemical treatment process, the quality of the effluent, the supply–demand balance, and the energy efficiency in the aeration process. Reducing energy consumption mainly includes: (1) controlling the constant of dissolved oxygen in the aerobic zone to prevent excessive aeration; (2) reducing the aerobic amount gradually according to the sewage treatment process; (3) setting the gradient to reduce the aeration amount (such as 35%, 30%, and 25%); and (4) adjusting the aeration amount according to the effluent ammonia nitrogen concentration [79]. When the activated sludge biochemical treatment process has a high load of COD, the main objective of aeration is to remove COD and carry out nitrification. So, the calculation of oxygen supply mainly considers these two biochemical processes. Through the precise aeration control biochemical section, the dissolved oxygen signal is used in order to access the control cabinet and turn to the wind pressure value by programming. Then the air pressure is used to control the aeration amount to achieve energy savings [80]. In addition, the unit energy consumption of the oxidation ditch process can also be reduced by using the brushing timing control. One of the key factors for blower control is to avoid aeration blower surge problems [81]. Controlling the blower outlet pressure is a necessary condition to solve the surge phenomenon and achieve DO automatic control, and the low DO and effluent ammonia nitrogen concentration control can achieve efficient automatic control and energy savings of the system. Meanwhile, precise aeration can also be achieved through Oxidation-Reduction Potential and pH-controlled wastewater treatment processes [82].

### 4.3. Nutrient Removal and Recovery

**Figure 6:**
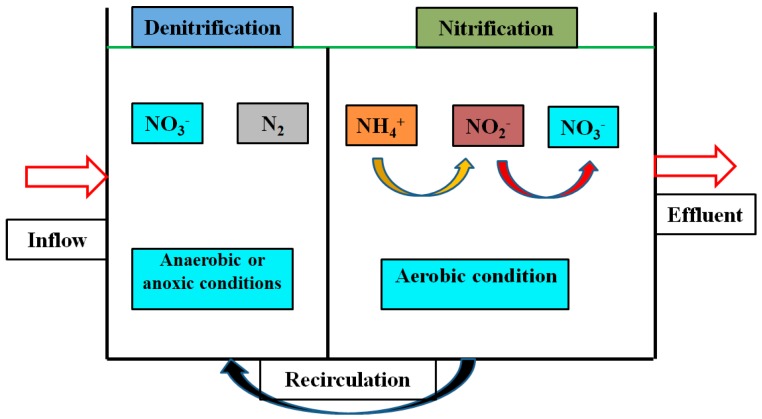
Biological nitrogen removal process.

With the in-depth study of the functional bacteria in the wastewater treatment process, new wastewater treatment processes that can realize energy savings from the technical point of view are gradually proposed. Those technologies mainly include wastewater denitrification processes based on short-cut nitrification, denitrifying, phosphorus removal processes, and anaerobic ammonium oxidation processes. Short-cut nitrification processes can save energy by 25% because ammonia nitrogen is only converted into nitrite instead of nitrate [83]. At the same time, denitrifying nitrite instead of nitrate can also reduce the demand for a carbon source for denitrification and strengthen wastewater nitrogen removal efficiency. For the anaerobic ammonium oxidation process, it is mainly applied to high ammonia nitrogen wastewater. Because only about 50% of ammonia nitrogen is oxidized to nitrite, less oxygen and energy is required in the process [84]. Recently, the feasibility of applying anaerobic ammonium oxidation to the main process of wastewater treatment process has also been considered, and a series of explorative research has been carried out [85]. Figure 6 illustrates the biological nitrogen removal process where the denitrification process and nitrification process are indicated separately. The denitrifying, phosphorus removal process mainly uses nitrate nitrogen as an electron acceptor to achieve simultaneous nitrogen and phosphorus removal, which save a large amount of aeration energy. Compared with the traditional enhanced biological phosphorus removal, the denitrifying phosphorus removal technology increases the carbon source utilization rate by 50%, saves 30% of aeration, and reduces sludge production by 50% [86].

The organic matter contained in the sewage is the energy carrier, so in addition to the energy savings of the sewage treatment, the realization of the energization of the wastewater can be considered [87]. In addition, the focus of energy recovery is to enhance the conversion of carbon-source organic matter into organisms in wastewater, and then to realize the energization of wastewater carbon sources through anaerobic fermentation. Research in this field needs to further develop the efficient reactors and the treatment technologies for the characteristics of sludge and wastewater [88].

### 4.4. Chemical Feeder

This method does not occupy a large proportion of energy consumption in wastewater treatment, but it takes a certain proportion in the stage of sludge disinfection, phosphorus removal, and sludge conditioning [89]. First of all, radiation sterilization techniques do not require high temperatures and pressures relative to other techniques. Irradiation has no obvious removal ability on heavy metals in the sludge. For safety reasons, most of this technology is still in the laboratory research stage [90]. In addition, biological disinfection technology is used by most sewage treatment plants without increasing the use of chemical agents. The process of removing phosphorus is complicated. In the chemical phosphorus removal process, the removal ability of polymeric iron or polymeric aluminum coagulant is better than that of ferric chloride and aluminum sulfate. The polymeric iron or polymeric aluminum coagulant requires less dosage when the same phosphorus removal capacity is obtained. In the process of excess sludge treatment, chemicals consumption in sludge conditioning is large and conditioner is expensive [91]. The sludge conditioning chemicals mainly includes coagulants and flocculants, and their functions are to flocculate the sludge particles into large flocs, which reduce the specific resistance and strengthen the dewatering property of the sludge [92]. Polyacrylamide and polyaluminum chloride are mainly used as coagulants and the diatomaceous earth, acid white clay, and lime can be used as flocculants, but they are difficult to biodegrade, which is not conducive to the subsequent [93]. High-performance, non-toxic, biodegradable natural polymer-modified coagulant with less dosage and high-dehydration efficiency such as fiber, polysaccharide, protein, and other polymer derivatives can be introduced to sludge conditioning in wastewater treatment plants [94].

### 4.5. Sludge Drying and Utilization

**Figure 7:**
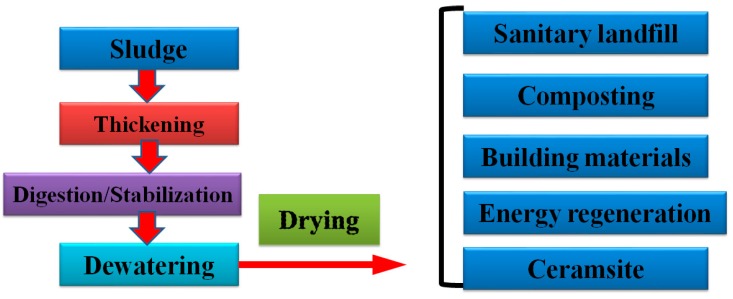
Flow chart of sludge treatment and disposal.

Heat-pump drying technology is a green drying technology that has the advantages of energy savings and low-environmental impact. Among them, the sewage source heat pump is highly feasible, and thus widely used. The principle of the sewage-source heat pump is to extract the low-level heat energy, store it in the urban sewage, and then convert it into a high-level energy source for outward output. The heat energy converted by the sewage-source heat pump can be output in various forms with a high heating temperature (up to 40 °C). In the sludge treatment process (Figure 7), the heat pump can be used for sludge drying before disposal. The sewage-source heat pump recycles the thermal energy in the urban sewage to dry the sludge while reducing the energy consumption of the wastewater treatment plant. Other low-carbon green energies, such as solar energy and geothermal energy, can also be used in the sludge drying process, such as by combining solar energy with a heat pump or geothermal energy with a heat pump to achieve energy diversification. This makes full use of the low-temperature solar collector to collect heat with the advantages of high heat-pump evaporation temperatures. The French Hans Amber Company assembles the heated floor in a solar drying system. In many solar drying systems, the heat pump extracts nearly 50% of the heat from the sewage and heats the water to about 60 °C to input the heated floor to assist the sludge drying.

## 5. Towards Energy-Positive Wastewater Treatment Plants

### 5.1. Biogas Power Generation Technology

Sludge is a by-product of sewage treatment and a new source of environmental pollution, which contains a large amount of organic matter. Therefore, sludge treatment and disposal are the final guarantee process for the whole sewage treatment. Sludge from sewage treatment can be used to prepare biogas and extract nitrogen, phosphorus, and organic matter. Biogas can be used to produce compressed natural gas or used for power generation while organic substances, such as nitrogen and phosphorus, are recovered in the form of struvite [95]. In addition, biogas can be used directly as a clean energy source for municipal gas or transportation fuel replenishment. Through high-temperature (35 °C) anaerobic digestion technology and biogas power generation technology, sludge can be converted into electric energy to compensate the power consumption of the sewage treatment plant. As a by-product for sewage treatment, sludge can be used to generate power and provide electricity for the operation of the sewage treatment plant. The generated biogas also can be converted into a certain economic value.

**Figure 8:**
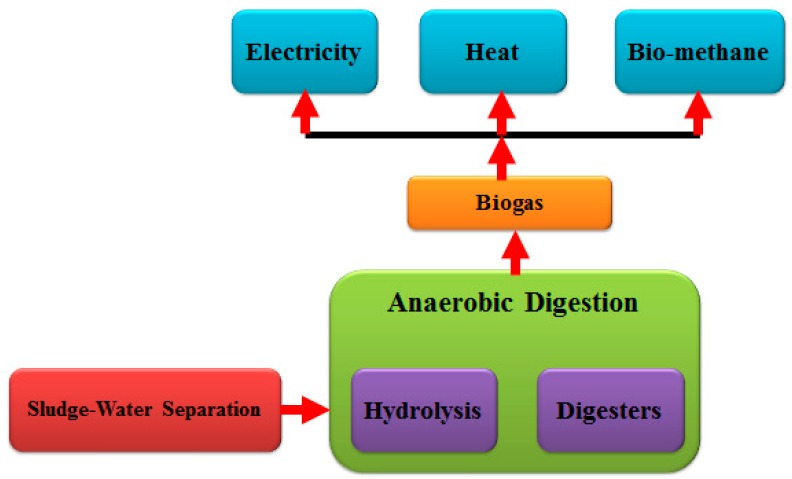
Main steps of anaerobic digestion in WWTPs.

Renewable energy includes biomass, solar energy, wind energy, etc. Biogas in biomass energy is an ideal renewable energy source [96]. Biogas is mainly produced by fermentation of industry, agriculture, and living waste. Through anaerobic fermentation of sludge, biogas can be produced in the wastewater treatment process, which has attracted wide attention [97]. Anaerobic fermentation can not only achieve sludge reduction, harmlessness, and stabilization, but also obtain resource recycling and produce clean energy biogas [98]. The methane content in biogas is high, which can be used to produce electricity by burning. The main steps for anaerobic digestion in WWTPs are shown in Figure 8. Anaerobic digestion of sludge is a biological treatment method for anaerobic microorganisms to decompose organic matter and produce biogas under anaerobic conditions [99]. The decomposition of organic matter is achieved by four stages of hydrolysis, acid production, hydrogen production, acetic acid production, and methanogenesis for producing methane. During the process, a large number of pathogenic bacteria and aphid eggs are killed. According to the literature [100], there are 50,000 sewage treatment plants in EU countries and the USA, and more than half of them use sludge anaerobic digestion, 66% in the United Kingdom and 58% in the United States in terms of the anaerobic digestion rate of sludge. Since the methane content in the digested biogas is 40–75% and the calorific value of methane is high (37,000 kJ/m^3^), so the calorific value of the digested biogas is generally 22,000 kJ/m^3^. In the anaerobic digester, the decomposition of 1 kg volatile suspended solids can produce 0.75–1.25 m^3^ of biogas, and the commonly used value is 0.8m^3^/kg corresponding to a heating value of 17,000 kJ/kg VSS.

Some of the general uses of biogas are shown in Table 1. Biogas is often used to heat the digester to maintain a system temperature at 35 °C (medium temperature digestion process) or 55 °C (high temperature digestion process) [101]. A biogas engine will be installed in certain places where digestion biogas is sufficient [102]. Biogas engines can be used to drive pumps, blowers or generators. When a suitable heat exchanger is used, the machine cooling water can be used as a heat transfer medium for pre-heating the digester sludge [103]. The waste heat after the biogas drives the engine can also be used for sludge drying or heating of common facilities. The hot steam generated in the boiler can be used in a sludge pasteurization process or a thermal hydrolysis process before digestion. Biogas can also be sold to power stations, factories or biogas utilization departments [104].

### 5.2. Photovoltaic Power Generation Technology

**Figure 9:**
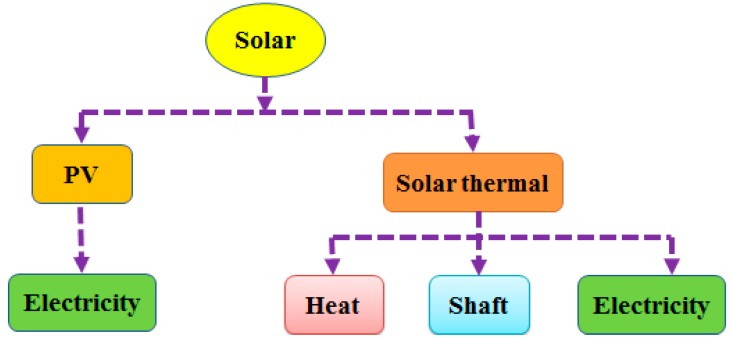
Pathways for solar energy utilization in wastewater treatment plants.

Solar photovoltaic power generation which has the remarkable advantages of cleanness, high efficiency, safety, and renderability has become one of the environmentally friendly alternative energy sources [105]. Developing photovoltaic power generation can reduce the pollution caused by the burning of mineral energy. Figure 9 shows the multiple pathways of solar energy applied in WWTPs. Sewage treatment plants require a large number of aeration tanks when treating sewage [106]. These require a lot of space in the plant area, and it provides the possibility to utilize the effective space on the structure to build a solar power generation facility to drive equipment or provide heat [107]. The wastewater treatment plant usually has a large-scale wastewater treatment tank which requires a large occupation area. The installation of solar photovoltaic panels has a unique advantage in space saving, so photovoltaic panels can be used in wastewater treatment plants which have limited land space [108]. The sewage treatment plant and the water supply plant have high electric load with large energy-consuming households. They operate continuously for 24 h with stale electric load. The power generated from the photovoltaic power stations can basically be used and consumed in wastewater treatment plants, which is in line with the “spontaneous use” mode. Among the direct production costs of wastewater treatment plants, electricity consumption accounts for more than 30%, so the technology to reduce costs and promote efficiency is a strong need for wastewater treatment plants [109]. The combination of photovoltaic power generation and wastewater treatment, and the implementation of contract energy management can further reduce the cost of wastewater treatment. Due to the needs of the process, some wastewater treatment tanks are covered to inhibit the growth of algae, and the construction of the grid above the pool surface is compatible with the capping requirements.

### 5.3. Sewage Water Source Heat Pump Technology

Urban wastewater is an excellent waste heat source. It has the characteristics that the wastewater temperature is higher than the atmospheric temperature in winter and lower than the atmospheric temperature in summer [110]. It is an ideal low-grade heat source for heat pumps. Sewage-source heat pumps are a system for extracting energy from urban domestic sewage [111]. According to the different states of sewage used by the system, sewage-source heat pumps can be divided into two types: one is a system that uses raw sewage as a source of cold heat; the other is a system that uses reclaimed water in the sewage treatment plant or water after secondary treatment as a source of cold heat [112]. The reclaimed water and water after secondary treatment is more suitable for a heat-pump system because of the improvement of water quality and the stability of water temperature. The main way of utilizing the thermal energy in the sewage treatment plant is to extract the heat energy from the reclaimed water and water after secondary treatment by the sewage source heat pump technology. One part of the energy is used to meet the internal heating demand of the sewage treatment plant, and the other part is used to transfer the heat energy to the heat station for urban building heating [113]. The use of sewage-source heat pump technology to extract heat energy from sewage has great potential for energy savings, and the extracted heat can be used for internal use in sewage treatment plants and building heating around sewage treatment plants [114]. It not only saves heat for the operation of the sewage treatment plant, but also facilitates the “carbon neutralization” operation mode of the sewage treatment plant. This achieves the performance of indirectly reducing carbon emissions and obtains a certain economic value of the heat output [115]. The sewage can be used as one of the heat sources for water-source heat pumps. The specific implementation method should be tailored to suit local conditions. The sewage-source heat pump technology can control the outlet water temperature according to the water treatment requirements. In addition, the sewage-source heat pump can also be operated intermittently according to the season.

## 6. Components for Integrating Green Energy with Energy–Efficient Water Technologies

### 6.1. Implementation Strategies and Action Plans

The overall energy-saving optimization operation strategy of the whole process is based on the rational deployment of wastewater treatment. From the energy balance analysis of the sewage treatment plant, the thermal energy of the sewage can be reduced with the process of the sewage treatment unit, which combined with the changing characteristics of various material components under various treatment conditions, rational distribution of the flow direction of the sewage energy substance can be achieved [116]. The overall guiding ideology of the whole energy-saving process and consumption–reducing operation strategy of urban sewage treatment plants are indicated as follows: The goal is of energy savings and consumption reduction in the whole process of sewage treatment with water quality guarantee as the constraint and the principle of “global optimal and local adaptation” [117]. The control components of the whole plant area are based on the multi-parameter intelligent operation control conditions with the formation of the optimal operation of energy savings and consumption reduction in the whole process of urban sewage treatment plants [118].

The daily operation management of the sewage treatment plant also has a very important impact on energy conservation and consumption reduction [119]. For example, in the daily operation management work, the pump frequency and the corresponding reflux ratio are timely performed and adjusted when the load of the sewage treatment system changes greatly. It is possible that the operating state of the sewage treatment system adapt to the load change. Thereby effectively avoiding energy waste caused by excessive aeration, which achieves the purpose of energy savings and consumption reduction effectively [120]. Therefore, the sewage treatment plant must develop a sound assessment and incentive system to manage employees according to the system, motivate employees’ work enthusiasm, and strengthen management norms, achieving a win–win situation for energy conservation and personal development for employees [121].

### 6.2. Instrument Control Automation (ICA) and Management Information System (MIS)

With the continuous improvement of the sewage treatment process and the increasing complexity of the project, traditional methods of manual operation and judgment by the operators are increasingly unable to meet the requirements of sewage treatment [122]. Therefore, the development and application of advanced automatic control systems is necessary. Automatic control systems are able to improve the automation of sewage treatment and monitoring, and can reduce the labor intensity of monitoring personnel and field operators. Furthermore, it also enhances the continuity of monitoring of sewage companies with obvious significance [123].

Compared to traditional manual operations, automated theory and control technology have become powerful tools and measures in wastewater treatment processes [124]. The application of self-control technology can save materials and energy more effectively, ultimately improving the stability of the system [125]. The instrumentation and automation of sewage treatment plants have become one of the major developments of the new water pollution control technology in the past decade. Whether it is the upgrading version of sewage treatment plants or the process design of new sewage treatment plants, process control has a great potential to be improved [126]. The control process improves the efficiency and effectiveness of process operation and control. The study of advanced dynamic models and the further application of simulators and actuators are an important factor in the continuous development of automatic control in wastewater treatment systems [127]. However, the current online sensing process in the closed loop control process is a barrier for the developing automatic control in the sewage treatment industry [128]. In recent years, many large and medium-sized sewage treatment plants have adopted a large number of online monitoring water quality analysis instruments to implement real-time monitoring of the water quality of the whole plant. Intelligent control has been applied to promote production process with high level of automation [129]. Most of the sewage treatment plants operating in China use simple process control in the complex treatment processes, resulting in some existing sewage treatment plants with poor operation results [130]. Some advanced technologies are difficult to be used in current sewage treatment plants, which lead to low operation efficiency [131].

### 6.3. Smart Technologies: Internet of Things (IoT)

Due to the dispersion of sewage treatment facilities, it will take a lot of manpower and material resources to carry out daily supervision of sewage treatment equipment [132]. It is necessary to effectively use the Internet of Things (IoT) technology to establish a point-to-point supervision mode from pollution source monitoring to sewage treatment and governance [133]. Therefore, it combines solar power supply, automation control, remote monitoring of the Internet of Things, and advanced treatment of sewage which establishes solar rural sewage treatment technology based on the IoT [134]. It automatically collects the sewage treatment system through automatic control and remote monitoring of the IoT. Operation data connected with a terminal management personnel mobile phone system may be a solution for the better management of a sewage treatment system; however, it is not yet in place. Internet of Things technology can effectively reduce the operation cost and improve the sewage treatment efficiency [135].

Nowadays, the aeration control mode mainly controls DO or ammonia nitrogen, and feedback is performed according to its concentration to achieve automatic control [136]. In the future, it is necessary to further study the refined control mode and method of wastewater treatment processes based on biochemical process simulation [137]. At the same time, optimization and development of new electrodes for control indicators also need to be studied. For different processes, establishing key energy consumption indicators and carrying out evaluation and optimization operations are also the objects that need to be researched in the future [138]. Due to the requirements of sewage treatment for nitrogen and phosphorus removal, the removal of organic carbon source in wastewater is mainly removed by denitrification and anaerobic processes. So, the demand of aerobic oxygen needs to be further evaluated and introduced for different processes into the process simulation control [139].

### 6.4. Comprehensive Performance Evaluation Programs

#### 6.4.1. Evaluation Approaches and Methodologies

At present, the detailed calculation of energy consumption in urban sewage treatment plants has been done. Table 2 summarizes the energy assessment methods for WWTPs. The evaluation methodology include analytic hierarchy process (AHP), life cycle assessment (LCA), fuzzy comprehensive evaluation method (FCE), specific energy consumption analysis method, and unit energy consumption analysis. Their features are also introduced in the table. Although relevant evaluation systems are gradually established, the topic explains whether the sewage treatment plant’s energy-savings is still attractive [140]. The evaluation of energy consumption for the sewage treatment plant is a comprehensive evaluation of multiple indicators and multi-objectives [141]. The feasible optimization plan for a specific problem and the decision-making becomes the focus of the experts’ research [142]. Through nearly 50 years of research, optimization methods and decision-making theories have been greatly developed, and some mature evaluation methods have gradually formed. These methods have successfully solved many practical problems in wastewater treatment plants [143].

#### 6.4.2. Key Performance Indicators (KPIs)

With the rapid development of China’s sewage treatment industry, the industry management and the operational performance (quality, efficiency, service level, and efficiency) in urban sewage treatment systems has become an important factor for urban operational safety, the water environment safety, the investment efficiency, and the impact of drainage [151]. The major issues of this sustainable development have received wide attention from society and enterprises. The implementation of operational performance management of the sewage treatment system can effectively promote the improvement of the operation quality and service level of the sewage treatment enterprise [152]. Through the performance appraisal of the sewage treatment system, the weakness in the production system operation and enterprise management are identified [153].

Constructing a performance evaluation index system for water systems and carrying out stylized performance evaluation can make comparison of wastewater supply performance in different periods and different enterprises. There are several indexes are used to evaluate the unit energy consumption of wastewater treatment plant, such as device indicators, specific energy consumption indicators, pollution emission indexes, recycling and utilization of resources, operation, and management and management and sustainability [153]. The detailed feature for each index is summarized in Table 3. This provides supervision tools and means for the government, and encourages water treatment enterprises to continuously improve for competitiveness [154].

### 6.5. Practical Implemention of Green Energy

In practice, renewable energy and green technology have become increasingly popular due to their potential to achieve the carbon neutral goal. Table 4 summarizes some WWTPs in the world which use green energy onsite, and the energy self-sufficiency for each case are listed. The WWTP in Eastern China occupies 716 acres, has a long-term treatment capacity of 800,000 ton/day. This WWTP applies solar photovoltaic power technology to generate electricity for onsite usage. It can produce 1.04 × 10^7^ kWh of electricity to satisfy the total energy demand of 1.23 × 10^7^ kWh. The Joint Water Pollution Control Plant with 95,000 ton/day treatment capacity uses sludge (91%) and food waste (9%) as feedstock to produce biogas. This resulting 1.75 × 10^8^ kWh/year electricity production makes the WWTP reach 97% energy self-sufficiency. Similarly, the Davyhulme plant in the UK with 63,000 ton/day treatment capacity can generate 96% of the total energy consumption through biogas power generation technology. For the Achill Island Central WWTP in Ireland, the implementation of wind power technology produces 7.6 × 104 kWh, which can satisfy 50% of annual energy use. The Aquaviva WWTP in France is a successful example for energy self-sufficiency. Through the application of sewage- and water-source heat-pump technology and solar power technology, the produced energy fully fills the demand of the process, providing 100% energy self-sufficiency.

## 7. Conclusions and Prospects

### 7.1. Combination Green Chemistry and Clean Production Technologies

The sewage treatment process is a process of purification by physical, chemical or biological treatment methods, so that sewage can meet the required water quality before discharging into a certain water body or being reused [159]. It can be seen that sewage treatment is a special production process, so the theory of cleaner production is also applied to wastewater treatment systems [160]. Clean production of sewage treatment systems includes saving electricity, heat and various chemicals used in the production process, reducing greenhouse gas emissions such as carbon dioxide and nitrous oxide, reducing the amount of sludge generated and improving the harmlessness and resource utilization of sludge. Correspondingly, the water quality from the sewage treatment system is further improved [161].

With the development of scale and resource utilization of sewage treatment, sewage treatment needs to reduce energy consumption, greenhouse gas emissions, and sludge deposal [162]. Therefore, clean production is significant in the efficient operation of sewage treatment plants, reducing resources and energy consumption and improving the quality of the surrounding environment, which gives full play to the economic, environmental, and social benefits of sewage treatment systems [163]. It also can promote the sustainable development of economic construction [164]. Therefore, the application of clean production methods in the energy savings and consumption reduction of sewage treatment systems has important theoretical significance for promoting the implementation of cleaner production [165].

### 7.2. Integration of Green Infrastructure and Ecologically Advanced Treatment Technologies

The sewage ecological engineering treatment technology refers to the application of ecological engineering principles and engineering methods to control sewage in a controlled manner [166]. The physical and chemical characteristics of the soil–plant–microbial composite system are used to treat the water and fertilizer resources in the sewage. The process technology for recycling and degrading degradable pollutants in sewage is a combination of sewage treatment and water resource utilization [167]. Commonly used sewage ecological engineering technologies include oxidation ponds, slow percolation, rapid percolation, surface flow, constructed wetlands, and soil percolation [168]. Due to the advantages of low investment, low operating cost and high efficiency of pollutant removal, the sewage ecological engineering treatment technology has been paid more and more attention in the application of small- and medium-scale sewage treatment [169].

(a) Sewage Land Treatment Systems

Sewage land treatment systems are an emerging ecological engineering technology used for sewage treatment [170]. This principle is to purify the pollutants in the sewage and utilize the sewage, nitrogen, and phosphorus resources through the chemical, biological, and physical fixation, then degradation of the soil–plant systems such as farmland, woodland, and depression [171]. We can divide the sewage land treatment system into five main process types according to the treatment target and the treatment object; those processes are slow percolation, rapid percolation, surface flow, wetland treatment, and underground percolation [172].

(b) Constructed Wetlands

Constructed wetlands are artificially constructed and regulated wetland systems that perform wastewater treatment through an optimized combination of physical, chemical, and biological interactions in their ecosystems [173]. Constructed wetlands generally consist of an artificial substrate and aquatic plants, such as cattails, reeds, and irises grown thereon to form a matrix-plant-microbial ecosystem [174]. As the sewage passes through the system, pollutants and nutrients are absorbed, converted or decomposed by the system, thereby purifying the water. Constructed wetlands are an open, dynamic, self-designed ecosystem that involve a multi-level food chain that forms a good internal material circulation and energy transfer function [175].

(c) Ecological Treatment System

The initial energy source of the ecological pond system is solar energy. Through the aquatic crops planted in the pond, the aquatic products and waterfowl culture are carried out, artificial ecosystems are established, and the biological treatment of sewage is completed through natural biochemical self-purification under natural conditions [176]. In the ecological pond treatment system, organic matter is degraded, and nutrients are released into a complex food chain, which make aquatic crops and aquatic products able to be harvested [177]. Domestic sewage and some organic industrial wastewater can be effectively treated by the ecological pond treatment system, which has the advantages of low investment, low operating cost, and simple operation management, with a good removal performance on organic matter and pathogens [178].

### 7.3. Optimization of the Water and Energy Nexus System

The energy consumption of the biological treatment unit in an urban sewage treatment plant accounts for about 60% of the total energy consumption of the process system, the pretreatment makes up about 25%, and the sludge treatment takes about 10% [179]. The power consumption mainly occurs in the sewage lifting system, the oxygen supply system of the biological unit and the sludge treatment system is the main linkage of energy savings and consumption reduction of a sewage treatment plant [180]. The energy consumption in the pretreatment stage is seriously wasted, with a large energy savings space. The main method is to scientifically select the pump and reasonably determine the pump head [181]. In the process design, the water in the mouth is changed to submerged outflow, reducing the head loss of the pipeline and the sewage lifting height [182]. In addition, due to the water inflow of the sewage plant changing with time and seasonal fluctuations, most of the time, the pumps cannot be operated efficiently, so the use of a variable frequency drive water pump is a very effective way to save energy [183].

The energy consumption in the biological treatment stage accounts for more than 50% of the total energy consumption, which is the most important energy-savings part for the wastewater treatment plant [184]. The main energy-saving methods are shown as follows: reduce pipeline resistance and pipeline leakage, install the blower as close as possible to the outer wall, use the blower frequency control technology; use DO control technology to control the air volume, use a low-resistance and anti-blocking micro-hole tube Aerator, arrange the aerator rationally, increase the aeration facilities at the front-end of the aeration tank, and reduce the amount of aeration at the back end [185].

With the increase of sludge discharge standards and the state’s emphasis on sludge treatment and disposal, the proportion of energy consumption in the sludge treatment section has increased [186]. Energy conservation mainly pays attention to the optimization of the concentration and dewatering process equipment, and the flocculant consumption is added to improve the dewatering performance of the sludge [187]. Therefore, the designer should accurately calculate the sludge production, water content, etc., and reasonably select the number of dewatering machines and its dewatering ability, it is best to determine the dosage of flocculant by experiment [188].

Sewage treatment plants generally have the problem that the actual water inflow is less than the design scale, so that most of the equipment in the sewage plant cannot be operated efficiently, resulting in a waste of energy consumption. In order to achieve energy savings and consumption reduction, the design of water quantity and quality is a problem that must be taken seriously [189].

## Figures and Tables

**Table 1:** The conventional utilization of digesting biogas.

Utilization Method	Equipment
Digestive tank heating	Boiler, heat recovery equipment, heat exchanger
Power generation	Biogas purification equipment, biogas generator
Building heating	Heat recovery equipment
Air conditioning	Heat recovery equipment
Sludge drying	Dryer, heat recovery equipment
Sludge pasteurization	Boiler, heat recovery equipment
Thermal hydrolysis	Boiler, heat recovery equipment
Methane sales	Biogas treatment equipment
Drive pump and blower	Biogas generator set
Combustion	Burner

**Table 2:** Assessment methods for energy conservation in wastewater treatment plants.

Evaluation Methodology	Introductions	References
Analytic hierarchy process (AHP)	AHP is a method of evaluation and decision-making that is often used in systems engineering. It decomposes the factors related to decision-making into goals, criteria, programs, and other levels. It relies on people’s subjective judgments to express and calculate in the form of quantity. Based on this, qualitative and quantitative analysis is carried out.	[144,145]
Life Cycle Assessment (LCA)	LCA, also known as structured system development method, is a popular information system development method. LCA is a tool that affects the entire life cycle environment until reuse or disposal, which evaluates a product from raw material extraction and processing to product production, packaging, marketing, use, product maintenance.	[146,147]
Fuzzy comprehensive evaluation method (FCE)	The fuzzy comprehensive evaluation method is a method of comprehensive evaluation of something using the basic theory of fuzzy mathematics. The evaluation method can transform the qualitative evaluation of something into quantitative evaluation according to the membership degree theory of fuzzy mathematics.	[148]
Specific energy consumption analysis method	The specific energy analysis method generally refers to the energy consumed by wastewater treatment plant per unit volume of wastewater, and the energy per unit volume is converted into electrical energy (kW × h/m^3^).	[149]
Unit energy consumption analysis	The unit energy consumption analysis method is based on the functions and energy consumption characteristics of each wastewater treatment structure of the wastewater treatment plant, and the wastewater treatment plant is divided into three main energy analysis units, and the energy consumption analysis and calculation are performed separately for each processing unit, and each analysis is performed independently. The energy consumption of the energy consumption unit, the main energy-consuming equipment, the energy consumption change law and the main energy consumption factor, and the energy consumption units are compared and analyzed to find the processing unit with the largest energy consumption and the equipment with the largest energy consumption in the unit.	[150]

**Table 3:** Evaluation indexes of unit energy consumption of wastewater treatment plants.

Device indicators	Equipment installed capacity, equipment operating capacity, equipment utilization, equipment maintenance rate, equipment operating efficiency, equipment maintenance frequency
Specific energy consumption indicators	Electricity consumption per ton water, chemicals consumption per ton water, chemicals consumption per ton dry sludge, the dry sludge produced by per ton wastewater, electricity consumption per ton dry sludge, electricity consumption for removing unit Chemical Oxygen Demand, electricity consumption for removing unit Total nitrogen, electricity consumption for removing unit Total phosphorus, water consumption per unit ton of wastewater, fuel consumption per unit ton wastewater
Pollution emission indexes	Raw water indexes, water output indexes, COD removal rate, ammonia nitrogen removal rate, TN removal rate, TP removal rate, dry sludge yield, COD, Biochemical Oxygen Demand, suspended solid, NH_3_–N, TN, TP, fecal coliform compliance rate, sludge treatment and disposal, enterprise plant boundary noise control, factory odor control, residents’ complaints
Recycling and utilization of resources	Water reuse, biomass energy utilization, solar energy utilization
Operation and management	Facility normal operation rate, facility load rate, automation control, operation management system implementation
Management and sustainability	Number of personnel, training fees, welfare fees, wages, system construction

**Table 4:** Practical implementation of green technology in WWTPs.

Country	Name/Position	Treatment Capacity	Green Technology	Energy Self-sufficiency	References
China	Eastern China	800,000 ton/day	Photovoltaic power generation technology	Saving 84% energy	[155]
UK	Davyhulme	63,000 ton/day	Biogas power generation technology	Saving 96% energy	[156]
Austria	Strass im Zillertal	228,000 ton/day	Biogas power generation technology	100% energy self-sufficiency	[157]
USA	Joint Water Pollution	95,000 ton/day	Biogas power generation technology	Saving 97% energy	[158]
Sweden	Stockholm	450,000 ton/day	Sewage, water-source heat-pump technology	5.97 × 10^8^ kWh energy production	[158]
